# Enhancing Engine Performance and Sustainability: Gold Nanoparticles and Machine Learning for Biodiesel Optimization in Compression Ignition Systems

**DOI:** 10.1021/acsomega.5c03571

**Published:** 2025-10-03

**Authors:** Amith Gadagi, Sneha Bandekar, Santhosh Paramasivam, Umesh Basanagouda Deshannavar, Natarajan Rajamohan, Chandrashekar Adake, Prasad G. Hegde, Gianluca Gatto

**Affiliations:** † Department of Mechanical Engineering, KLE Technological University’s Dr. M. S. Sheshgiri College of Engineering and Technology, Belagavi 590008, India; ‡ Department of Chemical Engineering, KLE Technological University’s Dr. M. S. Sheshgiri College of Engineering and Technology, Belagavi 590008, India; § Department of Electrical and Electronic Engineering, 3111 University of Cagliari, Cagliari 09123, Italy; ∥ Department of Chemical Engineering, Tatyasaheb Kore Institute of Engineering and Technology, Warananagar 416113, India; ⊥ Chemical Engineering Section, Faculty of Engineering, Sohar University, Sohar 311, Oman

## Abstract

Nanotechnology and machine learning are transforming energy systems by enhancing engine efficiency and sustainability. The integration of advanced nanomaterials, such as gold nanoparticles (AuNPs), with predictive modeling offers new opportunities for optimizing biodiesel performance in compression ignition (CI) engines. This study aims to develop and evaluate a novel biodiesel blend using 50% waste cooking oil and 50% Simarouba oil, enhanced with gold nanoparticles, and to apply machine learning for performance prediction and optimization in CI engines. Gold nanoparticles were synthesized from a plant extract and characterized by UV–visible spectrophotometry, confirming their presence at an absorption peak of 650 nm. Biodiesel blends (B20, B40, B60, and B80) were prepared and tested in a single-cylinder CI engine at various compression ratios (14:1, 16:1, and 18:1) and loads. Key performance parameters, including brake thermal efficiency (BTE) and brake specific fuel consumption (BSFC), were measured. An Extreme Gradient Boosting (XGBoost) machine learning model was trained on the experimental data to predict engine performance. The addition of AuNPs to biodiesel blends resulted in significant performance improvements: BTE increased by up to 6.57% for B20 at a 14:1 compression ratio, and BSFC decreased by up to 9.17% for B40 at a 16:1 compression ratio. The XGBoost model accurately predicted BTE and BSFC, with maximum errors of 4.17% and 3.53%, respectively. Gold nanoparticle-enhanced biodiesel blends offer improved CI engine performance and fuel efficiency. The use of XGBoost enables the reliable prediction of key performance metrics, reducing experimental costs and accelerating optimization. This integrated approach supports the development of sustainable, high-performance biofuels and advances the application of machine learning in energy systems.

## Graphical abstract

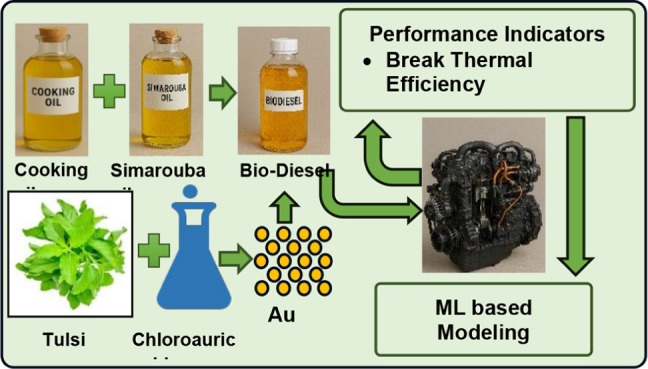

## Introduction

The increasing global demand for energy, coupled with the depletion of fossil fuel reserves and growing environmental concerns, has intensified the search for sustainable and eco-friendly alternatives to conventional petroleum-based fuels.^1,2^ Among the various renewable energy sources, biodiesel has emerged as a promising candidate due to its biodegradability, lower emissions, and potential for use in existing compression-ignition engines without requiring significant modifications.^3,4^ Biodiesel, typically produced through the transesterification of vegetable oils or animal fats, offers a renewable means to reduce dependence on finite fossil resources and mitigate greenhouse gas emissions.^5^ However, the widespread adoption of biodiesel is challenged by several factors, including competition with food resources, land use issues, and the variability in feedstock quality.^5–7^ Waste cooking oil and nonedible oils such as Simarouba oil have therefore gained attention as alternative feedstocks, providing a sustainable route for biodiesel production while addressing waste management concerns.^1,8,9^

Despite its environmental advantages, biodiesel faces several technical limitations when used as a direct replacement for diesel fuel in compression ignition engines. Notably, biodiesel typically exhibits higher viscosity, lower volatility, and a lower calorific value compared to mineral diesel, which can lead to incomplete combustion, increased brake-specific fuel consumption, and reduced engine efficiency.^10,11^ The combustion of biodiesel blends may result in higher emissions of nitrogen oxides, even as carbon monoxide, hydrocarbon, and particulate matter emissions are generally reduced.^10,12,13^ These challenges intensify when using biodiesel derived from waste cooking oil or nonedible sources, as the feedstock quality can vary significantly, impacting the physicochemical properties and overall fuel performance.^14^ Consequently, there is a pressing need to enhance the combustion characteristics and efficiency of biodiesel fuels to ensure their viability as a sustainable alternative for compression ignition engines.

Recent advances in nanotechnology have introduced novel approaches to improving the performance of biodiesel fuels. The addition of nanoparticles as fuel additives has shown considerable promise in enhancing combustion efficiency, reducing emissions, and improving fuel stability.^1,15^ Metal-based nanoparticles, such as those composed of aluminum oxide, cerium oxide, and zinc oxide, have been extensively studied for their catalytic properties, which promote more complete combustion and facilitate the oxidation of unburned hydrocarbons.^1,16,17^ Among these, gold nanoparticles have attracted particular interest due to their exceptional catalytic activity, stability, and ability to accelerate oxidation reactions at relatively low concentrations.^18^ The use of gold nanoparticles as fuel additives offers the potential to overcome many of the inherent drawbacks of biodiesel, including poor atomization, incomplete combustion, and increased fuel consumption.^19^ However, the high cost of gold remains a consideration, necessitating further research into the cost-effectiveness and practical benefits of such additives.

To address these challenges, researchers have investigated various methods for synthesizing and incorporating nanoparticles into biodiesel blends. Green synthesis approaches, utilizing plant extracts, have gained favor due to their environmental compatibility and ability to produce stable, well-dispersed nanoparticles. Synthesized nanoparticles are characterized using techniques such as ultraviolet–visible spectrophotometry, transmission electron microscopy, and dynamic light scattering to confirm their size, morphology, and stability.^20^ The prepared nanoparticles are then blended with biodiesel at optimized concentrations, often with the aid of surfactants and ultrasonication to ensure uniform dispersion and prevent agglomeration.^21^ The resultant nanofuel blends are evaluated in single-cylinder, four-stroke, direct-injection compression ignition engines under varying operating conditions to assess their impact on key performance parameters, including brake thermal efficiency and brake-specific fuel consumption.^22^ In addition to experimental evaluation, the adoption of advanced machine learning techniques, such as extreme gradient boosting, has enabled more accurate prediction and optimization of engine performance, reducing the need for extensive empirical testing.^23,24^

While numerous studies have demonstrated the benefits of adding metal oxide nanoparticles to biodiesel, there remains a paucity of research specifically focused on the use of gold nanoparticles synthesized via green methods and their impact on engine performance and emissions. Most existing research has concentrated on conventional feedstocks, with limited attention given to blends derived from waste cooking oil and nonedible oils such as Simarouba oil.^25,26^ The combined effects of gold nanoparticle enhancement and machine-learning-based performance prediction for these novel biodiesel blends have not been comprehensively explored. There is also a lack of systematic analysis regarding the stability of gold nanoparticle dispersions in biodiesel, the optimization of blending protocols, and the cost-effectiveness of such approaches in real-world applications.

In light of these considerations, this study aims to develop a novel biodiesel blend using equal proportions of waste cooking oil and Simarouba oil, enhanced with gold nanoparticles synthesized via a green, plant-extract-based method. The objectives of this research are 4-fold: (1) to characterize the physicochemical properties of the raw oils and the resulting biodiesel; (2) to synthesize and stabilize gold nanoparticles for use as fuel additives; (3) to evaluate the performance of gold nanoparticle-enhanced biodiesel blends in a compression ignition engine, focusing on brake thermal efficiency and brake-specific fuel consumption; and (4) to employ extreme gradient boosting machine learning models for the prediction and optimization of engine performance metrics. By addressing these objectives, this study aims to enhance the understanding of nanotechnology-assisted biodiesel optimization and lay the groundwork for the sustainable and efficient utilization of alternative fuel sources in modern engine systems.

## Materials and Methods

### Biodiesel Preparation

This study focused on evaluating engine performance using a gold-nanoparticle-based biodiesel blend, produced from waste cooking oil and Simarouba oil. To initiate the transesterification process, the oils were heated to 60 °C with a magnetic stirrer, ensuring uniform heating. Concurrently, a solution was prepared by dissolving 7 g of sodium hydroxide (NaOH) in 150 mL of methanol, to which 5 mL of sulfuric acid was then slowly added.

The transesterification reaction itself followed standard biodiesel production protocols, utilizing an oil-to-methanol molar ratio of 1:6.^27^ This mixture was combined with the 60 °C waste cooking oil and continuously stirred for 2 h at a constant speed of 600 rpm using a magnetic stirrer, ensuring uniform mixing and heat distribution while maintaining the temperature.

After the reaction, the mixture was allowed to settle for 24 h to facilitate the separation of glycerol. Following this phase separation, the crude biodiesel underwent purification through repeated water washing to remove residual catalysts, methanol, and soaps. This washing process continued until the wash water reached a neutral pH (approximately 7) and became clear, indicating the successful removal of impurities.^27–29^

The purity of the biodiesel was further confirmed by measuring the residual soap and catalyst contents, following ASTM D6751 standards. Finally, the biodiesel product was dried in a hot air oven at 105 °C until a constant weight was achieved, ensuring the complete removal of any residual water.^27,30^

### Physicochemical Properties of Raw Oils and Biodiesel

The physicochemical properties of waste cooking oil (WCO) and Simarouba oil (SO), as well as their respective biodiesel products (WCO-BD and Simarouba oil methyl ester, SOME), were determined according to the ASTM D6751 and EN 14214 standards. Parameters measured included density, kinematic viscosity, acid value, free fatty acid (FFA) content, methyl ester content, and composition of mono-, di-, and triglycerides.

Tables 1 and 2 present the detailed fatty acid composition and key physicochemical properties of various waste cooking oil samples, highlighting their significance as feedstocks for biodiesel, respectively. The free fatty acid profile in Table 1 emphasizes components such as oleic, linoleic, and palmitic acids, which play critical roles in fuel stability, oxidative resistance, and cold flow behavior. Table 2 summarizes essential quality parameters, including acid value, saponification value, viscosity, density, flash point, and moisture content, all of which influence the necessity for pretreatment, processing behavior, and eventual biodiesel yield.

**Table 1:** Free Fatty Acid Composition and Significance in Waste Cooking Oil Samples

	Content		
Free fatty acid	Waste cooking oil sample A	Waste cooking oil sample B	Significance
Oleic acid (%)	41.04 ^31,32^	43.67 ^31,33^	Oxidative stability
Linoleic acid (%)	17.98 ^31,32^	11.39 ^31,33^	Fuel stability
Palmitic acid (%)	31.88 ^31,32^	38.35 ^31,33^	Cold flow property

**Table 2:** Physicochemical Properties and Significance of Waste Cooking Oil Samples as Biodiesel Feedstocks

	Waste cooking oil value			
Property	Waste cooking oil sample C	Waste cooking oil sample D	Waste cooking oil sample E	Significance
Acid value (mg KOH/g)	1.86 ^31,34^	28.5 ^31,35^	35.4 ^31,36^	Indicates FFA.
Free fatty acid (%)	—	14.25 ^31,35^	18 ^31,36^	Determines pretreatment.
Saponification value (mg KOH/g)	181.25 ^31,34^	175.87 ^31,35^	234.71 ^31,36^	Soap formation risk.
Viscosity (mm^2^/s)	42.01 ^31,34^	46.97 ^31,35^	—	Processing flowability.
Density (kg/m^3^)	—	908 ^31,35^	916 ^31,36^	Energy content.
Flash point (°C)	234 ^31,34^	223 ^31,35^	—	Storage safety.
Moisture content (%)	0.1 ^31,34^	—	0.136 ^31,36^	Transesterification yield.

Table 3 presents a comparative overview of the key fuel properties of conventional biodiesel, diesel, and Simarouba oil biodiesel. Parameters such as cetane number, ash content, flash point, and kinematic viscosity are considered to evaluate the fuel performance, combustion quality, and storage safety of Simarouba biodiesel in comparison with established fuels.

**Table 3:** Comparative Physicochemical Properties of Biodiesel, Diesel, and Simarouba Oil Biodiesel

**Property**	**Biodiesel**	**Simarouba oil biodiesel**	**Diesel**
Ash content (%)	–	0.50% ^31,37^	100 maximum^31,38^
Carbon residue (%)	0.05 maximum^31,38^	10.00% ^31,37^	0.2 maximum^31,38^
Cetane number	47 minimum^31,38^	–	46 ^31,38^
Density at 15 °C (kg/m^3^)	880 ^31,38^	865 ^31,37^	820–860 ^31,38^
Flash point (°C)	≥130 ^31,38^	165 ^31,37^	60–80 ^31,38^
Higher heating value (MJ/kg)	42.65 ^31,38^	–	46.48 ^31,38^
Kinematic viscosity at 40 °C (mm^2^/s)	1.9–6.0 ^31,38^	4.68 ^31,37^	2.0–4.5 ^31,38^
Pour point (°C)	–15 to −16 ^31,38^	14.2 ^31,37^	–35 to −15 ^31,38^

Table 4 provides the physicochemical properties of diesel and biodiesel blends, both with and without gold nanoparticle additives. It highlights the variations in density, viscosity, calorific value, and flash point with increasing biodiesel concentration. It assesses the influence of nanoparticles on these properties, thereby offering valuable insight into fuel handling and combustion behavior.

**Table 4:** Physicochemical Properties of Diesel and Biodiesel Blends without Nanoparticles

Sl No	Blends	Density (g/cc)	Kinematic viscosity (stokes)	Relative viscosity	Calorific value (kJ/kg)	Flash point (°C)
Properties of biodiesel						
1	DIESEL	0.8194	0.0278	4.1500	42000	65
2	B20	0.8270	0.0305	5.5344	41244	68
3	B40	0.8371	0.0309	5.6157	40488	74
4	B60	0.8488	0.0324	5.7566	39732	87
5	B80	0.8587	0.0333	5.8629	38976	128
6	B100	0.8790	0.0425	6.3659	38220	172
Properties of biodiesel with nanoparticles (Au)						
1	B20	0.8340	0.0313	5.5648	41244	68
2	B40	0.8382	0.0311	5.6567	40488	74
3	B60	0.8491	0.0326	5.7599	39732	87
4	B80	0.8620	0.0335	5.8682	38976	128
5	B100	0.8850	0.0430	6.3820	38220	172

The data indicate that transesterification significantly reduces viscosity and acid value while increasing methyl ester content, confirming successful biodiesel production. The addition of gold nanoparticles to biodiesel blends further improves fuel properties, maintaining density and viscosity within optimal ranges and supporting enhanced combustion and engine performance. The second table highlights the superior quality of biodiesel enhanced with gold nanoparticles, underscoring its suitability for engine applications.

Fuel property analysis (Tables 1, 2, and 4) confirms that gold nanoparticles enhance biodiesel by reducing the kinematic viscosity by 1.2–3.7% compared to non-nano blends, while maintaining a calorific value equivalent to that of base biodiesel and optimizing density for improved atomization. These align with nanofuel studies showing that viscosity reduction directly lowers BSFC, while stable calorific value sustains BTE.^39,40^

### Gold Nanoparticle Synthesis

Gold nanoparticles can be synthesized through chemical, physical, and biological methods. Chemical methods include citrate reduction (Turkevich), thiol-based two-phase synthesis (Brust–Schiffrin), and seed-mediated growth. Physical methods involve laser ablation, sonochemical synthesis, and thermal decomposition. Biological methods utilize plant extracts, micro-organisms, or enzymes for environmentally friendly synthesis.^40,41^

To prepare the plant extract for reducing gold ions, 10 g of thoroughly washed, fresh tulsi (*Ocimum sanctum*) leaves were combined with 100 mL of sterile, double-distilled water in a 200 mL Erlenmeyer flask, maintaining a mass ratio of 1:10 (w/w), as recommended in the literature.^42,43^ The leaves were then shade-dried for 3 days, milled, and sieved to collect a powder with a volume surface mean diameter of approximately 120 μm (passing through a 212-μm screen and retaining on a 75-μm screen) for consistent extraction efficiency.^44^ The tulsi leaf powder was soaked in the same volume of double-distilled water and boiled at 60 °C for 60 min to obtain the extract.

A 1 mM chloroauric acid (HAuCl_4_) solution was prepared by dissolving 0.339 g of HAuCl_4_ in 100 mL of water. For gold nanoparticle preparation, 10 mL of the plant extract was added to 90 mL of the HAuCl_4_ solution and stirred for 90 min at a temperature below 50 °C. The formation of gold nanoparticles was confirmed by a color change from dark brown to light brown. The mixture was ultrasonicated for 60 min to dry the nanoparticles. After centrifugation at 14,000 rpm for 10 min, the wet nanoparticles were separated, dried, and collected.^45–47^

Biodiesel was prepared by blending Simarouba oil and waste cooking oil in a 1:1 mass ratio, with each contributing 50% by weight. For the incorporation of gold nanoparticles at a concentration of 20 ppm (weight per weight), they were initially mixed with a small volume of biodiesel and cetyltrimethylammonium bromide surfactant. This mixture was then added to the main biodiesel batch and subjected to ultrasonication at 200 W and 40 kHz for 45 min at room temperature, ensuring uniform dispersion. The average particle size of these gold nanoparticles was determined to be 25 ± 5 nm, as measured by dynamic light scattering using a Malvern Zetasizer Nano ZS. The resulting blend was used immediately following its preparation, with no observation of phase separation. The stability of the gold nanoparticle dispersion was further confirmed through visual inspection and periodic sampling over 48 h; no sedimentation was detected, indicating excellent colloidal stability.

### Fourier Transform Infrared Analysis

Gold ion reduction was confirmed by UV–vis spectroscopy (200–800 nm, 1 nm resolution, 2 nm interval) using a BIOMATE16Q spectrometer (Figure 1). FTIR spectroscopy, typically within the 4000–400 cm^–1^ range, is utilized to identify functional groups involved in the synthesis and stabilization of gold nanoparticles. Characteristic vibrational peaks for O–H (∼3433 cm^–1^), C–H (∼2924/2840 cm^–1^), C=O (∼1633 cm^–1^), and C–O/C–C (∼1047 cm^–1^) confirm the presence and role of organic capping agents.^48–50^ FTIR spectroscopy (4000–0 cm^–1^, 4 cm^–1^ resolution) with KBr mixing was performed using a Nicolet Summit LITE FTIR Spectrometer (Figure 2). For nanoparticle dispersion, 0.02 g of Au nanoparticles and CTAB surfactant were stirred ultrasonically in 500 mL of biodiesel for 45 min.

**Figure 1:**
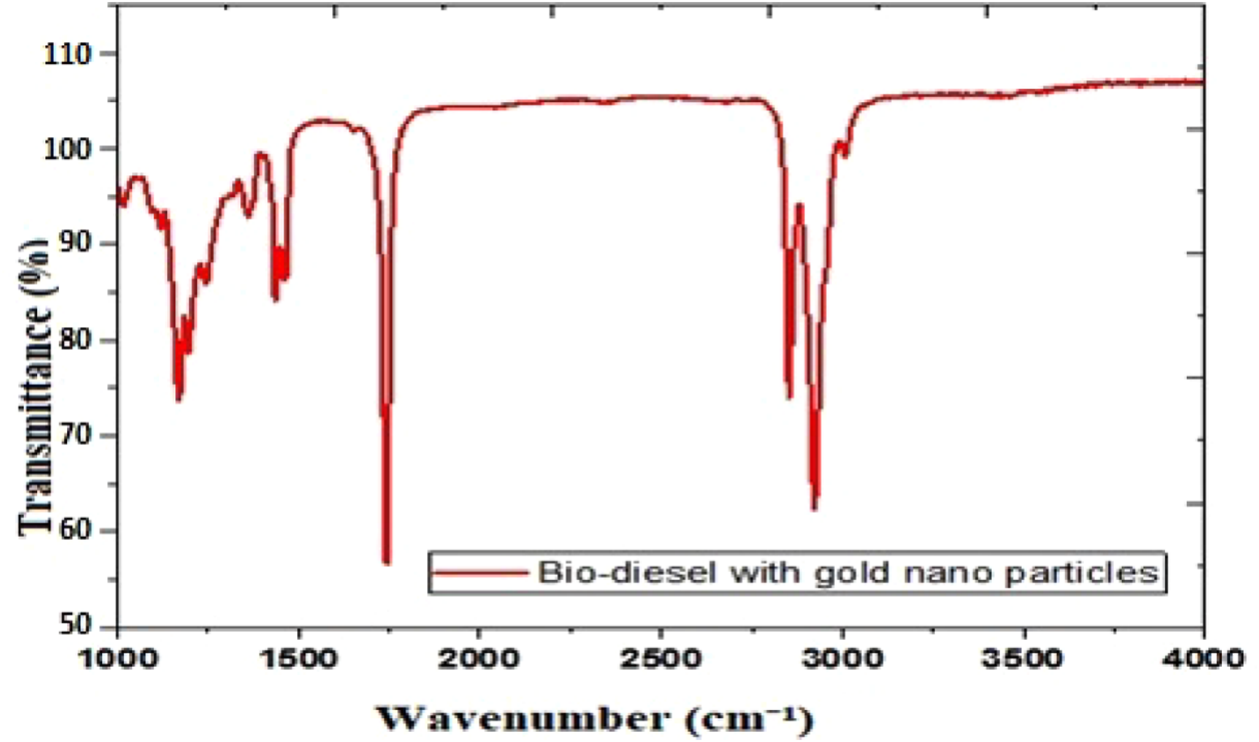
FTIR spectra of gold nanoparticle-blended biodiesel.

**Figure 2:**
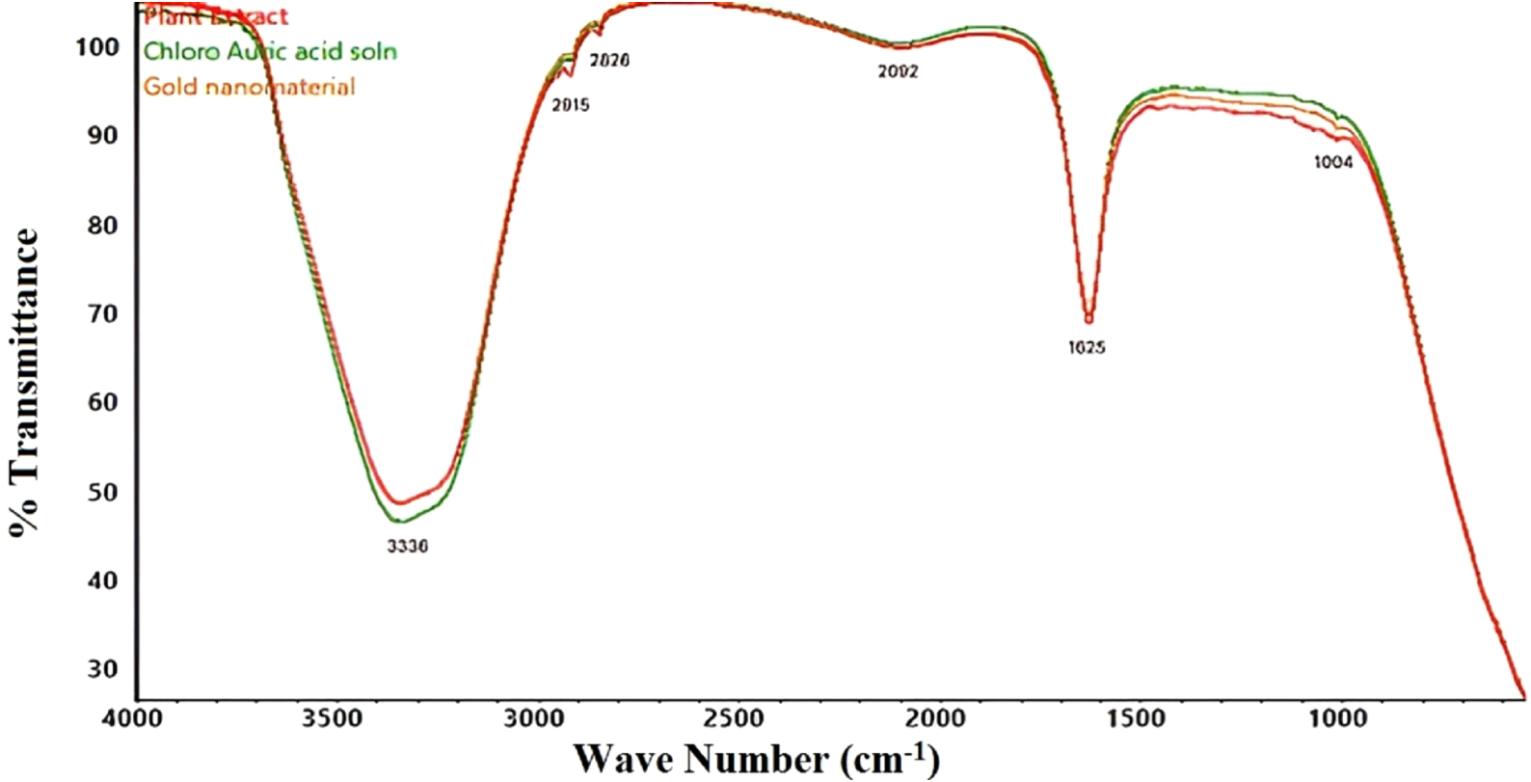
FTIR spectra of a plant extract, chloroauric acid solution, and gold nanoparticles.

The FTIR spectrum (Figure 1) exhibits strong absorption (3000–3500 cm^–1^), indicating N–H or O–H stretching vibrations (associated with amines, alcohols, and water). Sharp peaks around 2900 cm^–1^ suggest alkane stretching. Intense absorption near 1700 cm^–1^ represents the C=O stretching vibration (common in carboxylic acids, ketones, esters, and aldehydes). Fingerprint region peaks (1500–1000 cm^–1^) indicate C–C, C–N, and C–O vibrations. The sample contains alkane (−CH), carbonyl (C=O), ether/amine (C–O–C/–NH), and hydroxyl (−OH) functional groups. Peaks at 2918 cm^–1^ suggest the presence of hydroxyl groups in phenols and alcohols, indicating the reduction of gold nanomaterials.^51–53^

The FTIR spectrum in Figure 2 compares gold nanoparticles, chloroauric acid, and a plant extract. A broad peak at 3336 cm^–1^ indicates O–H stretching, suggesting the presence of hydroxyl groups. Peaks at 2826 and 2915 cm^–1^ (C–H stretching) confirm the presence of organic compounds. The 2092 cm^–1^ peak corresponds to C=N or C=C stretching, while strong peaks at 1625 and 1004 cm^–1^ indicate carbonyl stretching. Similar spectra of gold nanoparticles and plant extracts suggest the retention of functional groups after synthesis. Differences in the chloroauric acid spectrum confirm the reduction of gold ions. FTIR analysis shows that plant phytochemicals act as reducing and stabilizing agents.^54^

The UV–vis absorption spectra shown in Figures 3 and 4 are obtained to investigate the presence of absorbing species and key absorption peaks. Studies have demonstrated the use of UV–vis spectroscopy (350–800 nm, 1 nm resolution) to confirm the formation of gold nanoparticles and study their optical properties.^48,50^ A peak at approximately 210 nm represents Pi-to-Pi electronic transitions, which suggest the presence of nucleic and amino acids. The peak at approximately 270 nm represents n-to-Pi electronic transitions present in C=O compounds. The UV–vis spectrum exhibits differences in absorption bands at 270 and 210 nm, indicating nanoparticle interactions and the presence of carbonyl groups.

**Figure 3:**
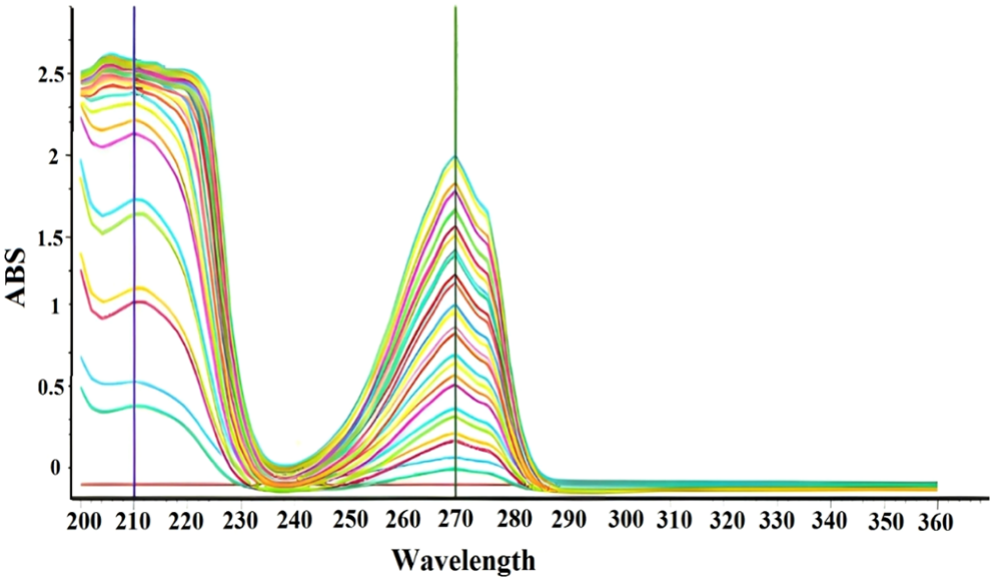
UV–vis absorption spectra.

**Figure 4:**
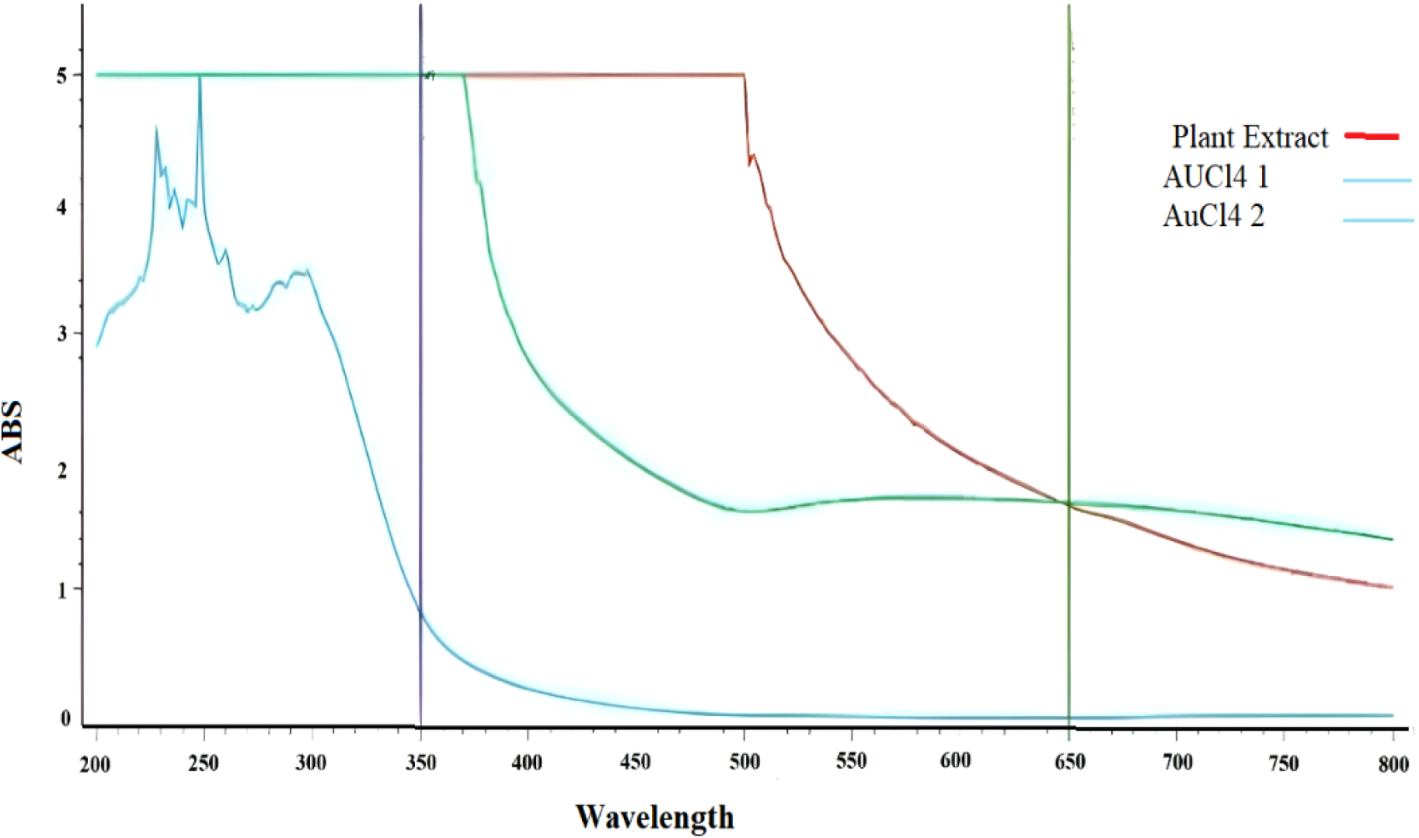
UV–visible absorption spectra of gold nanoparticles, AuCl_4_-1 and AuCl_4_-2.

Figures 3 and 4 display UV–vis absorption spectra (200–800 nm) analyzing the absorption properties of AuCl_4_ and plant extracts. The plant extract shows strong absorption at 350 nm (ABS = 5.0) and moderate absorption at 650 nm (ABS = 1.657). AuCl_4_ (sample 1) exhibits moderate absorption at 350 nm (ABS = 0.824) and minimal absorption at 650 nm (ABS = 0.003), confirming the AuCl_4_ complex. Strong absorption at 350 nm (ABS = 5.0) and increased absorption at 650 nm (ABS = 1.678) indicate the formation of gold nanoparticles. The differences in absorption spectra between AuCl_4_ sample 1 and sample 2 are attributed to variations in synthesis conditions, such as reactant concentration, temperature, or mixing duration, which affect nanoparticle size and distribution.^55,56^ This formation was further confirmed by a color change (from brown to yellow) and UV spectrophotometer measurements, as gold nanomaterials typically exhibit maximum absorption within the 550–650 nm wavelength range.

### Engine Setup

Experiments were conducted on a single-cylinder, four-stroke, direct-injection Kirloskar compression ignition diesel engine. The engine was tested under varying loads, ranging from 0 to 12 kg (rated load), at 3-kg intervals. Biodiesel blends (B20, B40, B60, B80) were evaluated at 23° before top dead center with compression ratios of 14:1, 16:1, and 18:1. The blends B20, B40, B60, and B80 refer to blends containing 20, 40, 60, and 80% biodiesel by volume, respectively. All engine tests were conducted with identical injection parameters, including injection timing (23° before top dead center), injection pressure, and duration, to ensure comparability between fuels with and without gold nanoparticles. All engine tests were conducted with identical injection parameters, including injection timing (23° bTDC), injection pressure, and duration, to ensure comparability between fuels with and without gold nanoparticles.

The Kirloskar Engine test rig features a single-cylinder, four-stroke, water-cooled diesel engine with a bore and stroke of 87.5 mm × 110 mm. The engine operates at 1500 rpm and delivers a rated power of 3.5 kW. It offers an adjustable compression ratio ranging from 12:1 to 18:1, with the current configuration set at 17.5:1. The complete experimental setup includes all necessary instrumentation for a comprehensive performance evaluation and data collection.

The engine’s compression ratio was precisely adjusted to 14:1, 16:1, and 18:1 using a calibrated variable compression ratio mechanism, which allowed for modification of the clearance volume via a threaded cylinder head adjustment, verified with a dial gauge. An electronically controlled eddy current dynamometer applied increments of 1 kg up to the maximum rated load. All experiments were conducted at a constant engine speed of 1500 rpm, with loads of 3, 6, 9, and 12 kg applied using the dynamometer. All performance measurements, including brake thermal efficiency and brake-specific fuel consumption, were accurately recorded by using integrated sensors and a digital data acquisition system.

### Extreme Gradient Boosting (XGBoost)

Extreme Gradient Boosting, commonly referred to as XGBoost, is a machine learning technique used for classification and prediction tasks. This algorithm is a supervised learning method that utilizes gradient-boosting decision trees to achieve high prediction accuracy. Because of the use of gradient-boosting decision trees, the XGBoost model can be learned more quickly. The computations in the Extreme Gradient Boosting model happen in parallel and sequentially. XGBoost can capture and handle nonlinear data patterns during model training. In this machine learning technique, every weak learner corrects itself, thus giving rise to a new learner during every iteration. The loss functions of this method are minimized by using the most common gradient descent algorithm. The regularization term involved in this model avoids the possibility of overfitting in the predictions. In this work, the dataset required for XGBoost is generated through experiments, where for every biodiesel blend containing Au nanoparticles, the CI engine is run at loads of 3, 6, 9, and 12 kg for compression ratios of 14:1, 16:1, and 18:1. The training dataset includes all data points from Table 5 except those with Sl. Nos. 3, 6, 17, 23, 28, 34, 42, and 48, which were used for testing.

**Table 5:** Experimental Results of BTE and BSFC for Different Biodiesel Blends, Compression Ratios, and Loads

Sl. No.	Biodiesel blend	Compression ratio	Load (kg)	BTE (%)	BSFC (kg/kW h)
1	B20	18:1	3	9.8462	0.8724
2	B20	18:1	6	16.8462	0.5141
3	B20	18:1	9	19.9231	0.4947
4	B20	18:1	12	22.0000	0.3956
5	B20	16:1	3	10.3846	0.8382
6	B20	16:1	6	16.6154	0.5283
7	B20	16:1	9	19.6923	0.4463
8	B20	16:1	12	21.8462	0.3956
9	B20	14:1	3	9.6923	0.8981
10	B20	14:1	6	16.0769	0.5511
11	B20	14:1	9	19.3846	0.4406
12	B20	14:1	12	20.2308	0.4326
13	B40	18:1	3	11.0767	0.8732
14	B40	18:1	6	17.2317	0.5146
15	B40	18:1	9	20.3405	0.4927
16	B40	18:1	12	22.8243	0.3902
17	B40	16:1	3	10.5295	0.8390
18	B40	16:1	6	16.6067	0.5268
19	B40	16:1	9	19.7153	0.4488
20	B40	16:1	12	21.1835	0.4073
21	B40	14:1	3	9.9828	0.8951
22	B40	14:1	6	16.2948	0.5512
23	B40	14:1	9	19.4814	0.4585
24	B40	14:1	12	21.0280	0.4220
25	B60	18:1	3	10.3216	0.8665
26	B60	18:1	6	16.1575	0.5590
27	B60	18:1	9	19.8266	0.4814
28	B60	18:1	12	20.5727	0.4224
29	B60	16:1	3	10.2713	0.8882
30	B60	16:1	6	16.1072	0.5590
31	B60	16:1	9	19.4740	0.4689
32	B60	16:1	12	21.2781	0.4286
33	B60	14:1	3	9.7170	0.9255
34	B60	14:1	6	15.8050	0.5683
35	B60	14:1	9	19.2725	0.4689
36	B60	14:1	12	20.5223	0.4379
37	B80	18:1	3	10.1597	0.8922
38	B80	18:1	6	16.5188	0.5418
39	B80	18:1	9	19.7197	0.4725
40	B80	18:1	12	20.9934	0.4210
41	B80	16:1	3	10.3199	0.9040
42	B80	16:1	6	16.5722	0.5596
43	B80	16:1	9	19.0769	0.4814
44	B80	16:1	12	20.9401	0.4329
45	B80	14:1	3	10.1596	0.9011
46	B80	14:1	6	16.6258	0.5625
47	B80	14:1	9	19.5590	0.4637
48	B80	14:1	12	21.4219	0.4329

## Results and Discussion

Brake thermal efficiency and specific fuel consumption are the most critical performance indicators characterizing any compression ignition engine. Thus, the aforementioned performance indicators are investigated by running the diesel engine fueled by biodiesel made up of Simarouba and waste cooking oil, as well as the biodiesel mixed with gold nanoparticles in blends such as B20 and B40.

The comparative results for brake thermal efficiency and brake-specific fuel consumption at loads of 3, 6, 9, and 12 kg were selected for detailed discussion. The maximum load of 12 kg was chosen because it represents the most demanding operating condition, where differences in fuel performance are most noticeable.

### Brake Thermal Efficiency

The enhanced brake thermal efficiency (BTE) of biodiesel produced from waste cooking oil (WCO) enhanced with AuNPs stems from improved combustion. Gold nanoparticles (AuNPs) enhance combustion efficiency through their catalytic properties. AuNPs promote faster, more consistent flame spread and thorough fuel burning by accelerating oxidation reactions and reducing the activation energy required for ignition. This process increases heat release, resulting in higher cylinder pressures and temperatures, and ultimately enhances engine thermal efficiency.

Catalytic activity accelerates oxidation and reduces the ignition activation energy, promoting rapid and consistent flame propagation and complete combustion. As a result, heat release, cylinder pressures, and temperatures increase, leading to improved engine thermodynamic efficiency. In addition, AuNPs modify fuel properties such as viscosity and surface tension, which enhance atomization and spray characteristics. Improved atomization promotes better air-fuel mixing, creating a homogeneous charge and efficient combustion. Consequently, the combined effects of these mechanisms significantly increase the level of BTE, positioning AuNP-enhanced biodiesel as a promising alternative to conventional diesel.

Figure 5 shows the comparison plot of maximum BTE for the B20 biodiesel blend and the B20 biodiesel blend with Au nanoparticles for different compression ratios of 18:1, 16:1, and 14:1. At maximum load, BTE values corresponding to the B20 biodiesel blend with Au nanoparticles for the compression ratios of 18:1, 16:1, and 14:1 are 22.001%, 21.889%, 21.879%, respectively. In contrast, for the cases without Au nanoparticles, maximum BTE values are 20.86%, 21.08%, and 20.53%, respectively. It can be inferred from Figure 6 that the maximum BTE values increased by 5.46%, 3.84%, and 6.57% with the addition of Au nanoparticles. At high engine loads, gold nanoparticles not only reduce ignition delay but also promote more complete and rapid combustion during the main combustion phase, resulting in a higher rate of heat release near TDC, which improves the conversion of fuel energy to useful work, thereby increasing BTE. The concentration of AuNPs was optimized to avoid excessive advancement of combustion phasing, and no significant increase in negative compression work was observed.

**Figure 5:**
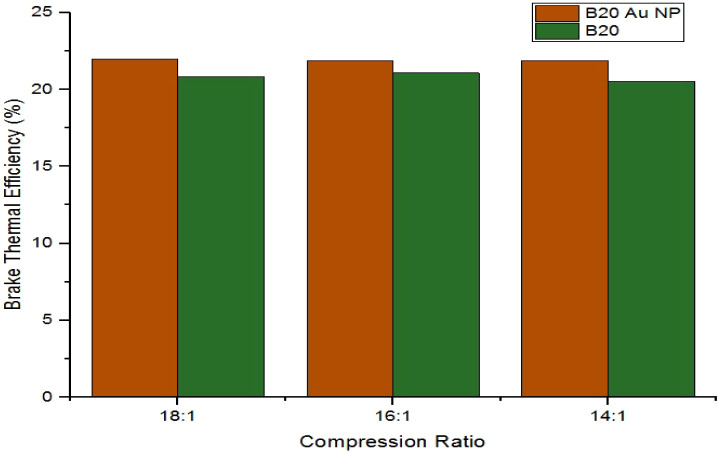
Comparison plot of maximum BTE for B20 and B20 AuNPs.

**Figure 6:**
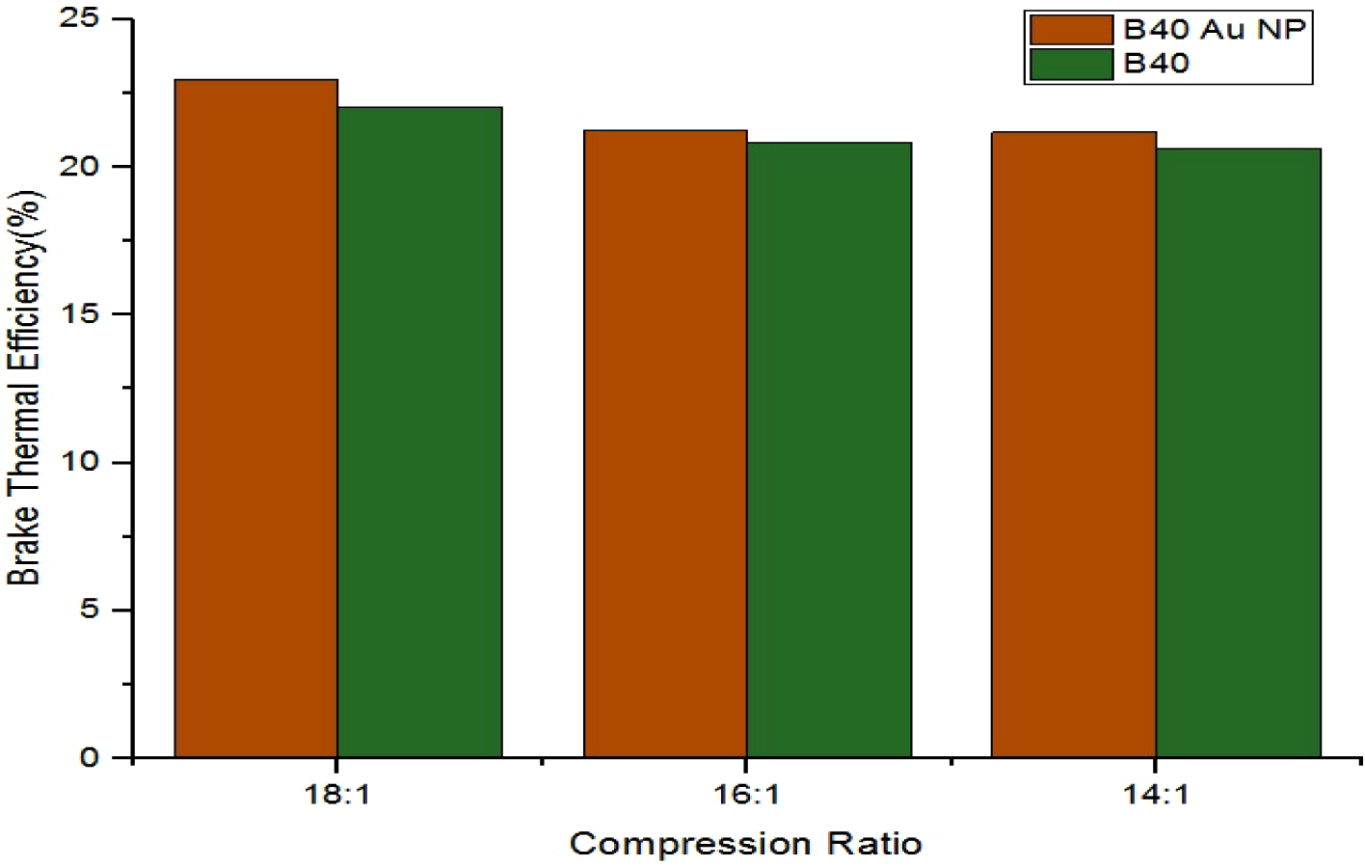
Comparison plot of maximum BTE for B40 and B40 AuNPs.

The comparison plot of maximum BTE for the B40 biodiesel blend and the B40 biodiesel blend with AuNPs for the different compression ratios of 18:1, 16:1, and 14:1 is shown in Figure 6. The maximum BTE values corresponding to the B40 biodiesel blend with Au nanoparticles for the compression ratios of 18:1, 16:1, and 14:1 are 22.96%, 21.23%, and 21.18%, respectively. In contrast, for the cases without Au nanoparticles, the maximum BTE values are 22.04%, 20.86%, and 20.65%, respectively. Figure 6 shows that the maximum BTE values increased by 4.17%, 1.77%, and 2.56% with the addition of Au nanoparticles.

Gold nanoparticles (AuNPs) improve BTE and align with the consistent trend reported for various other nanoparticles, including Al_2_O_3_, ZnO, CeO_2_, TiO_2_, MgO, and CuO.^57–64^ The inferred mechanism of enhanced combustion efficiency via catalytic activity is strongly supported by other studies, which explicitly attribute catalysis to MgO, ZnO, and CuO^60,64^ and report improved combustion metrics, such as higher heat release rates,^65,66^ increased cylinder pressures,^67^ and shorter ignition delays,^68,69^ across different nanoparticle types. Significant BTE increases are reported for ZnO (11.7%),^64^ CeO_2_ (up to 12.64%),^63^ MgO (8.3%),^60^ Al_2_O_3_ (3.6–3.8%),^57,62^ and CuO (up to 14.6%).^64^ The observed improvements in brake thermal efficiency (BTE) of about 1.8–6.6% fall within this expected range. However, some metal oxides might enhance performance further through extra effects like supplying oxygen^70–72^ or improving fuel atomization,^73–75^ which could explain the higher improvements reported. The direct link between these improved combustion characteristics and resulting BTE gains is well-supported^59,60,62,64^

### Brake-Specific Fuel Consumption

Integrating gold nanoparticles into waste cooking oil biodiesel blends offers a multifaceted approach to enhance combustion efficiency and reduce emissions in compression ignition engines. The technical improvements stem from the unique physicochemical properties of AuNPs and their interactions with the fuels. Primarily, AuNPs serve as potent catalysts, facilitating enhanced oxidation reactions during combustion. The catalytic activity promotes a more complete conversion of fuel hydrocarbons into carbon dioxide and water, releasing more energy per unit of fuel consumed. Such improved chemical reactions directly reduce brake-specific fuel consumption, as less fuel is required to achieve the same power output. Additionally, the high surface area-to-volume ratio of AuNPs ensures more significant interaction with fuel molecules, accelerating reaction kinetics and improving overall combustion efficiency.

Figure 7 plots the maximum BSFC of a CI engine running on B20 biodiesel (with and without Au nanoparticles) at compression ratios of 18:1, 16:1, and 14:1. With nanoparticles, the maximum BSFC values were 0.870, 0.838, and 0.896 kg/kW h, respectively. Without nanoparticles, these values were 0.876, 0.870, and 0.924 kg/kW h. The addition of Au nanoparticles reduced BSFC by 0.68%, 3.67%, and 3.03% at 18:1, 16:1, and 14:1 compression ratios, respectively.

**Figure 7:**
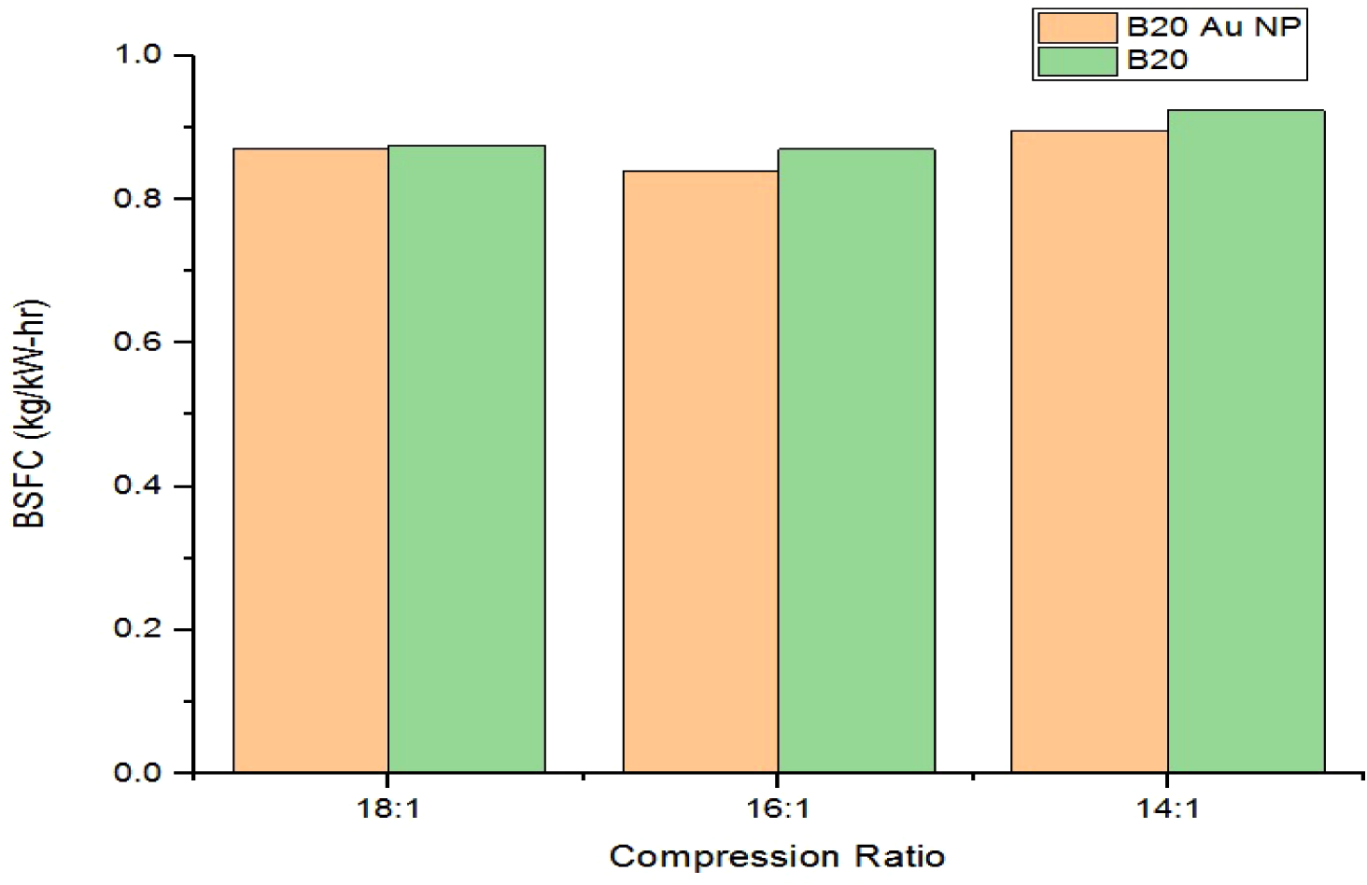
Comparison plot of maximum BSFC for B20 and B20 AuNPs.

The comparison plot of maximum BSFC values for the B40 biodiesel blend and the B40 biodiesel blend with AuNPs for the different compression ratios of 18:1, 16:1, and 14:1 is given Figure 8. The maximum BSFC values corresponding to the B40 biodiesel blend with Au nanoparticles for the compression ratios of 18:1, 16:1, and 14:1 are 0.861 kg/kW h, 0.842 kg/kW h, and 0.890 kg/kW h, respectively. In contrast, for the cases without Au nanoparticles, the maximum BSFC values are 0.853, 0.927, and 0.924 kg/kW h, respectively. It is clear from Figure 8 that, only for the compression ratio of 18:1, there is a negligible increase of 0.93% in maximum BSFC. However, for the compression ratios of 16:1 and 14:1, the maximum BSFC values decreased by 9.17% and 3.67% with the addition of Au nanoparticles, respectively.

**Figure 8:**
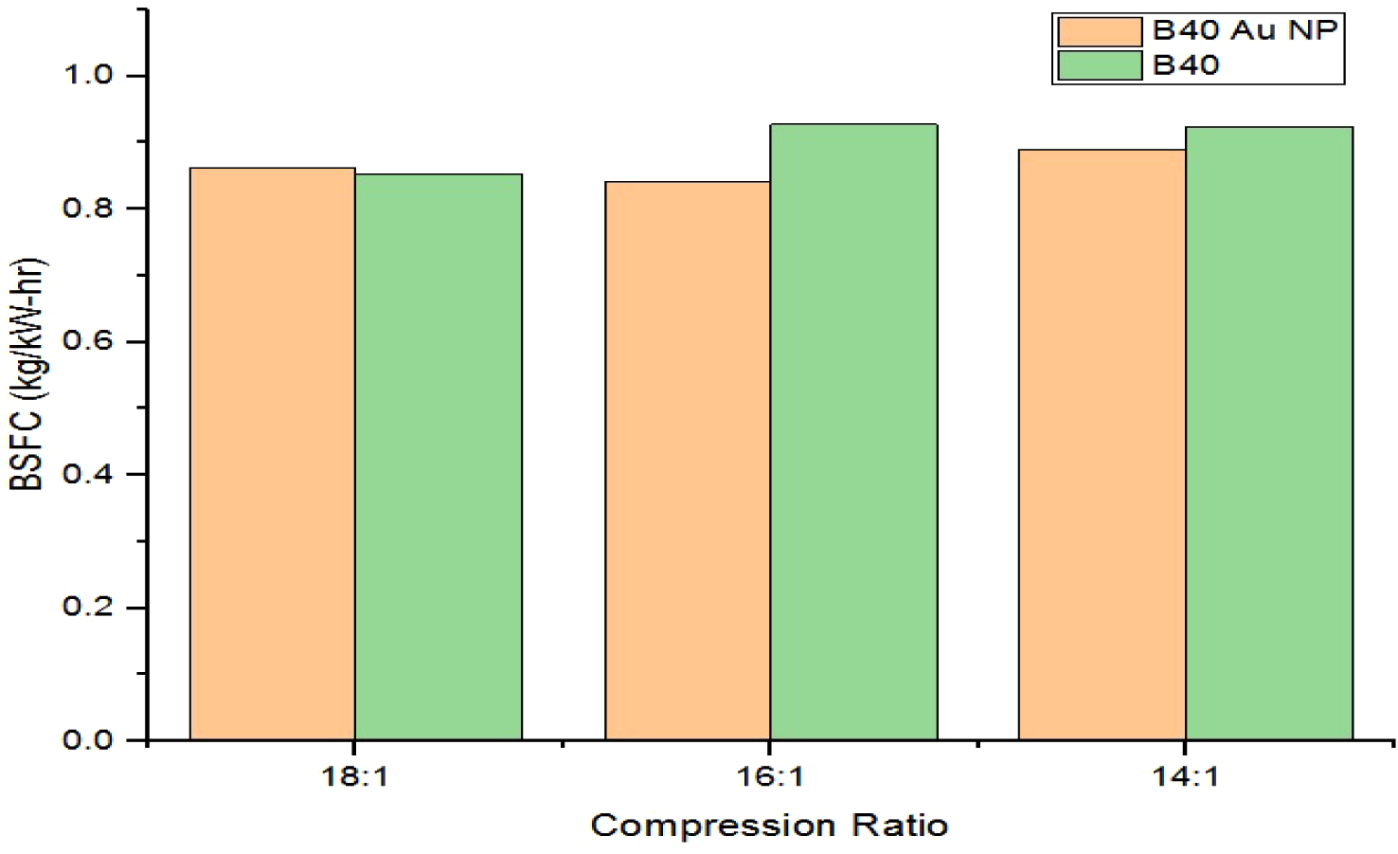
Comparison plot of maximum BSFC for B40 and B40 AuNPs.

BSFC reduction observed with AuNPs is consistent with the general effects of other nanoparticle fuel additives.^57–62^ The proposed mechanism, involving enhanced combustion efficiency due to catalytic action that releases more energy per unit of fuel, logically leads to lower fuel consumption (BSFC) and is supported by several studies.^59,62,64^ Numerous studies document a reduction in the ratio of Brake Specific Fuel Consumption to Brake Specific Energy Consumption (BSFC/BSEC): Al_2_O_3_ showed reductions of up to 3.7%,^62^ ZnO by 1.67–6.3%,^58,60^ CeO_2_ enhanced BSFC and reduced BSEC by 10%,^59,63^ TiO_2_ reduced BSFC by 5.9%,^60^ and CuO achieved reductions of up to 8%.^64^ The BSFC reductions observed in this study (∼0.7–3.7%) are comparable to the reported values.

### Cost-Effectiveness Considerations

Gold nanoparticles are indeed more expensive than other commonly used nanoadditives. However, in our study, AuNPs were used at very low concentrations (typically 20–100 ppm), which minimizes the impact on overall fuel cost. The observed improvements in brake thermal efficiency (BTE) and reductions in brake-specific fuel consumption (BSFC) mean that less fuel is required for the same power output, partially offsetting the cost of the additive.^76,77^ Literature also indicates that nanoparticle additives, even when costly per unit mass, can yield significant fuel savings and a positive return on investment due to enhanced combustion and reduced engine wear.^78^ For large-scale or commercial applications, the economic feasibility of gold nanoparticles depends on the balance between additive cost, efficiency gains, and the potential for nanoparticle recovery or recycling. For research and specialized applications, their superior catalytic performance justifies their use.

Comparison of the XGBoost values of brake thermal efficiency for the training and testing datasets with the corresponding experimental values in Figures 9 and 10, respectively. We find that the maximum prediction errors for training and testing instances of BTE are 0.05% and 4.17%, respectively.

**Figure 9:**
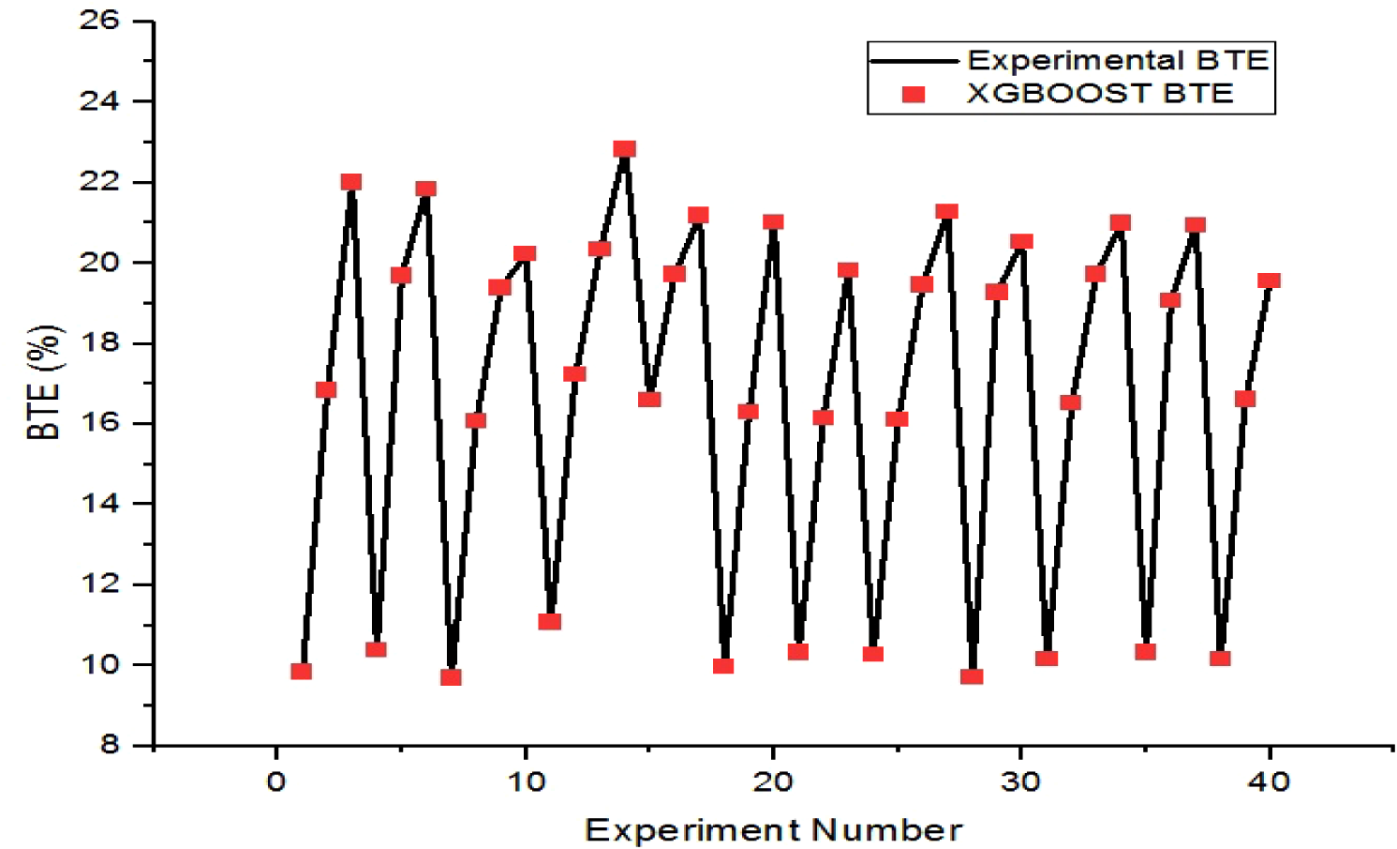
Comparison plot of XGBoost BTE and experimental BTE for the training set.

**Figure 10:**
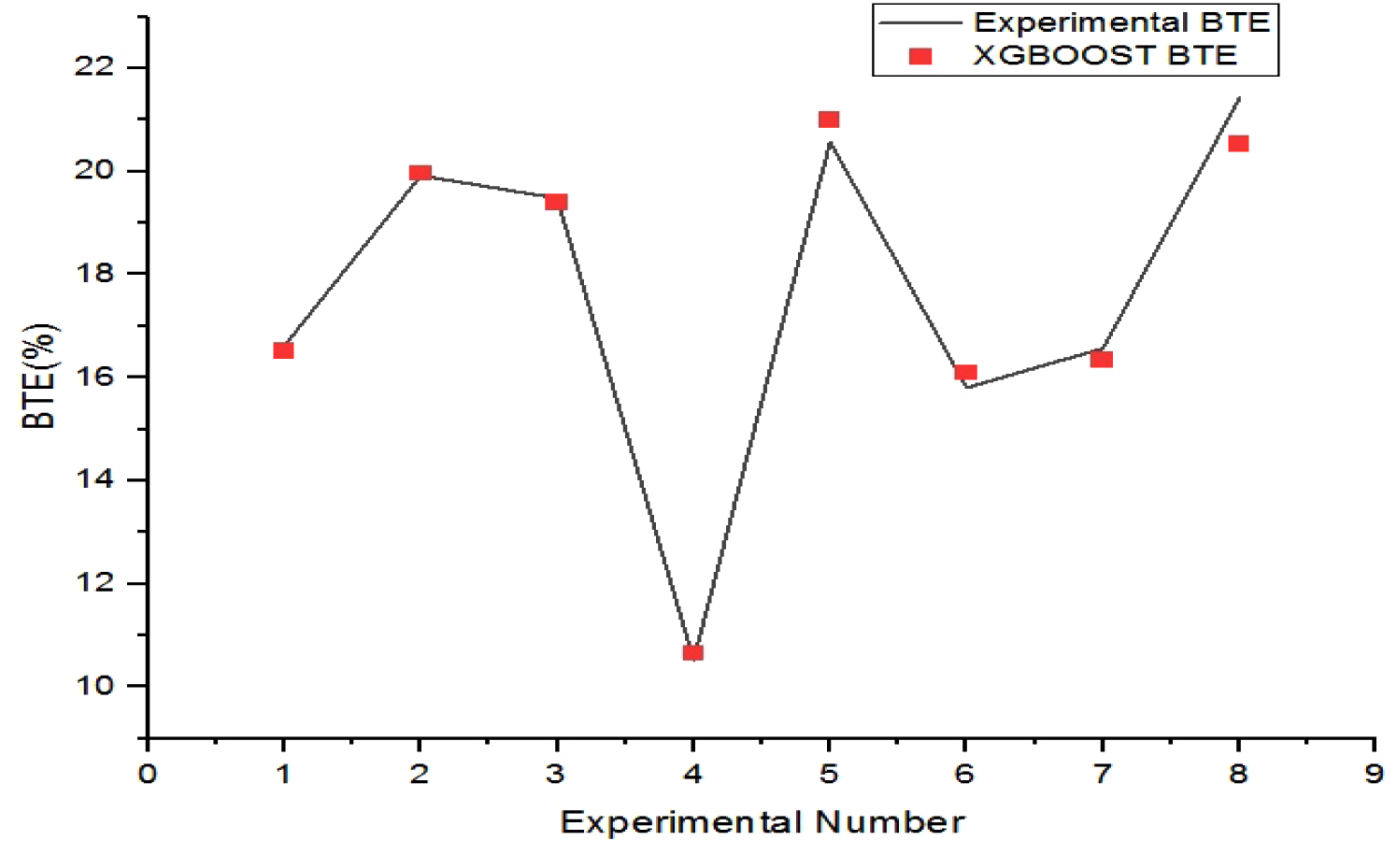
Comparison plot of XGBoost BTE and experimental BTE for the testing set.

The XGBoost-predicted values of brake-specific fuel consumption are compared with the respective experimental values for the training and testing datasets in Figures 11 and 12, respectively. The maximum error of prediction in the training and testing instances of BSFC is found to be 0.85% and 3.53%, respectively.

**Figure 11:**
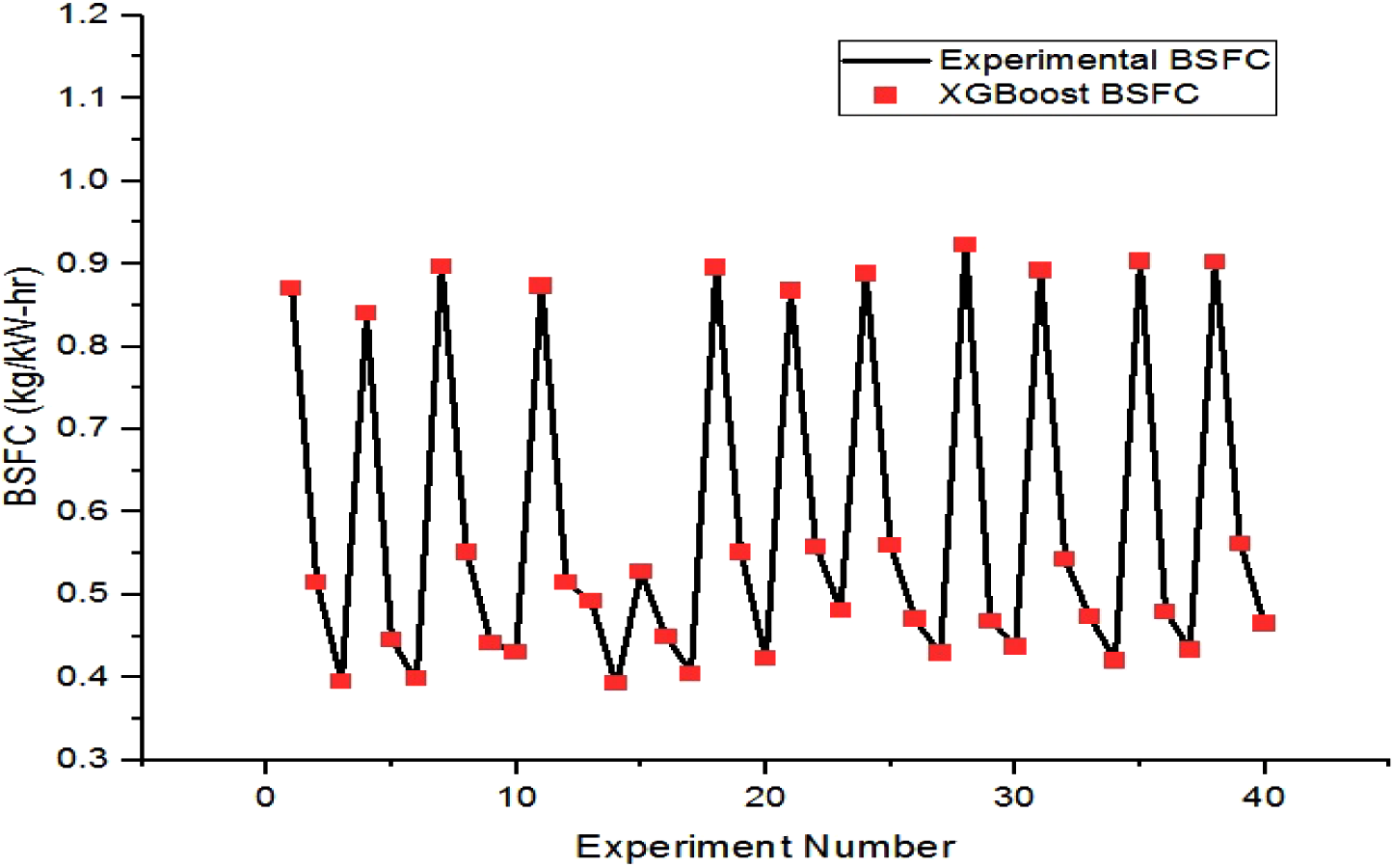
Comparison plot of XGBoost BSFC and experimental BSFC for the training set.

**Figure 12:**
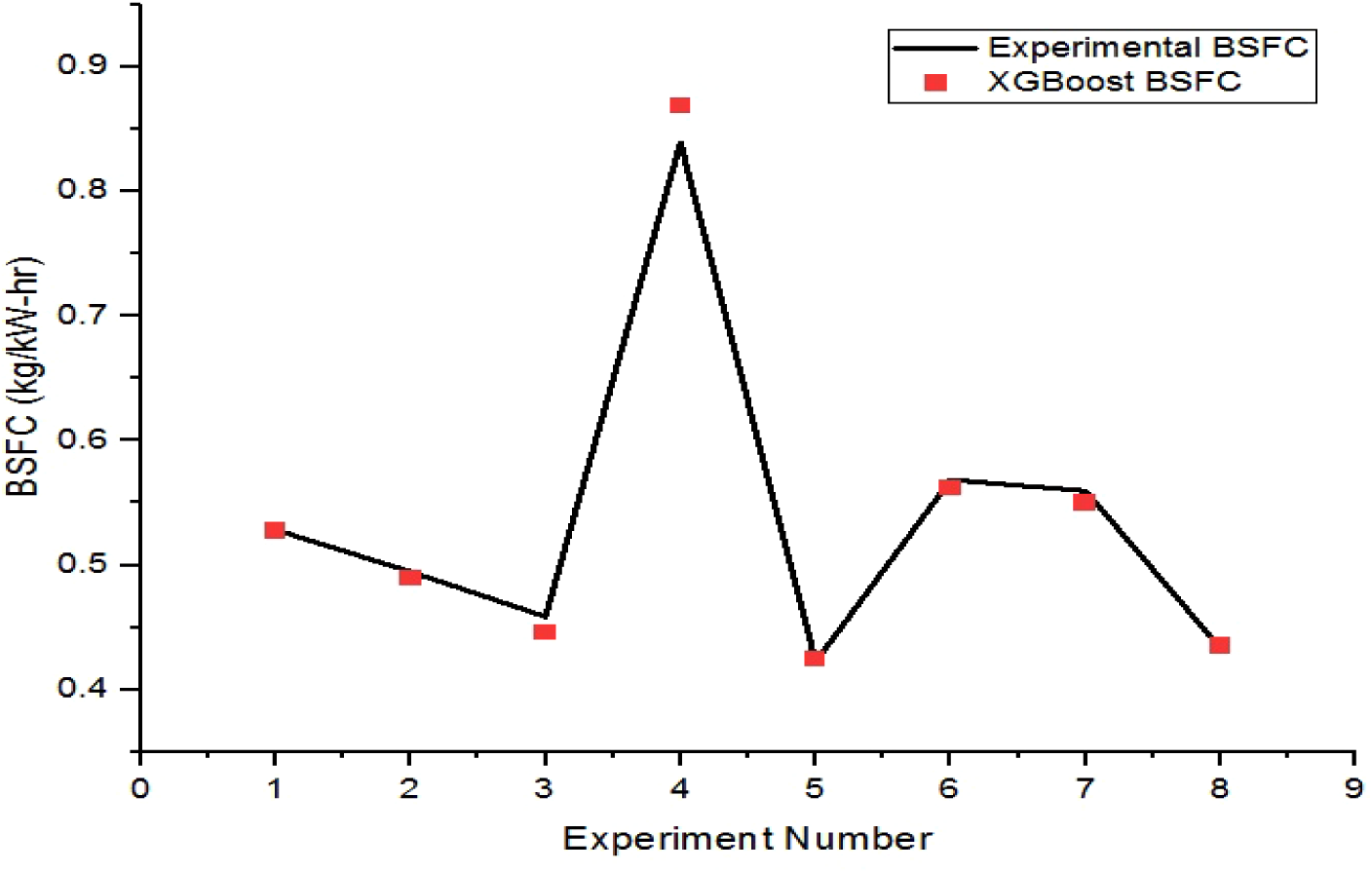
Comparison plot of XGBoost BSFC and experimental BSFC for the testing set.

The 2D contour plots of XGBoost BTE and BSFC for training and testing instances are compared with the corresponding experimental contour plots in Figures S1–S4. Both the XGBoost and experimental contours appear to be a good match.

XGBoost for predicting BSFC and BTE aligns with the extensive application of diverse machine learning techniques, like Gaussian Process Regression (GPR), Artificial Neural Network (ANN), Boosted Regression Trees (BRT), XGBoostRegressor (XGBR), and Advanced Machine Learning (ML) models, including Artificial Neural Networks (ANN), K-Nearest Neighbors (KNN), Extreme Gradient Boosting (XGB), and Random Trees (RT), which were employed for predictive analysis in similar prediction tasks.^79–82^ Predictive models, including hybrid GPR-FNN architectures, ensemble methods like Random Forest (RF), and Artificial Neural Networks (ANN), effectively predict engine performance parameters. These models achieve high accuracy, yielding test *R* ^2^ values of 0.9921 for BSFC (GPR-FNN), 0.9887 for BTE (GPR-FNN), 0.97 for BTE (Wide ANN), and 0.9620 for BSFC (RF), which validates the low prediction errors achieved in the XGBoost prediction.

Figures S1–S4, as given in the Supporting Information, provide a detailed comparison between XGBoost model predictions and experimental results for both brake thermal efficiency (BTE) and brake-specific fuel consumption (BSFC). Figure S1 shows BTE for the training dataset, with subplot (a) displaying XGBoost predictions and subplot (b) showing experimental values. Figure S2 presents the BTE comparison for the testing dataset in the same format. Figures S3 and S4 similarly compare BSFC for the training and testing datasets, respectively, with (a) representing XGBoost and (b) experimental results. The close alignment between predicted and experimental values across all figures demonstrates the model’s robustness and predictive accuracy.

The comprehensive statistical analysis presented in Table 6 demonstrates the excellent predictive capability of the XGBoost model.

**Table 6:** Statistical Performance Metrics of the XGBoost Model for Predicting BTE and BSFC

	Brake thermal efficiency	Brake specific fuel consumption
MSE	0.1430	0.0001
*R* ^2^	0.9860	0.9910
MAPE	0.0150	0.0141
MAE	0.2700	0.0085
RMSE	0.3700	0.0122

The statistical performance metrics presented in Table 6 demonstrate the predictive accuracy of the XGBoost model. The high coefficient of determination values (*R* ^2^ = 0.986 for brake thermal efficiency and *R* ^2^ = 0.991 for brake specific fuel consumption) indicate that the model successfully captures over 98% of the variance in both output parameters. The low error metrics, with MAPE values below 1.5% and RMSE values of 0.37 and 0.0122, respectively, confirm minimal prediction errors and excellent agreement between model predictions and experimental observations. These results validate the reliability and robustness of the XGBoost approach for accurately predicting engine performance parameters.

### Environmental Impact and Emission Analysis

This study primarily focused on engine performance metrics (BTE and BSFC), as well as the environmental impact of gold nanoparticle (AuNP) additives, particularly their effect on emissions, which remains a critical consideration for sustainable fuel development. Although direct emission measurements were not conducted in this work, extensive literature indicates that the addition of nanoparticles to biodiesel blends typically promotes more complete combustion, resulting in substantial reductions in carbon monoxide (CO) and unburned hydrocarbon (HC) emissions due to enhanced catalytic oxidation and improved air–fuel mixing.^83–85^ Gold-based catalysts have been commercialized for emission control, with documented reductions in CO, HC, and particulate matter of up to 30% in diesel engines.^84,85^ The effect on nitrogen oxides (NOx) is complex. While increased combustion efficiency can elevate in-cylinder temperatures, potentially increasing NOx, some studies report that nanoparticles can also lower NOx due to uniform combustion and reduction in residence time at high temperatures.^83,84^ Future work will focus on comprehensive emission characterization to further validate the environmental benefits of AuNP-enhanced biodiesel blends.

## Conclusions

In this study, a novel biodiesel blend consisting of 50% waste cooking oil and 50% Simarouba oil was developed and enhanced with gold nanoparticles. The presence of these nanomaterials was confirmed through color identification and ultraviolet–visible spectrophotometry, which revealed a peak absorption at 650 nanometers. The performance of B20 and B40 biodiesel blends containing gold nanoparticles in a compression ignition engine was evaluated with a focus on brake thermal efficiency (BTE) and brake-specific fuel consumption (BSFC). The results showed significant improvements, including a 6.57% increase in BTE for B20 blends at a compression ratio of 14:1 and a 5.46% increase for B20 blends at a compression ratio of 18:1, respectively. Additionally, adding Au nanoparticles reduced BSFC by 3.67% for B20 and 9.17% for the B40 blends. An XGBoost machine learning model was employed to enhance further predictive capabilities, which accurately forecasted BTE and BSFC with maximum errors of 4.17% and 3.53%, respectively. The present work is intended to showcase the catalytic potential of AuNPs and provide a scientific benchmark for future studies, including those focused on cost optimization and alternative nanoadditive strategies. This innovative approach shows that gold nanoparticle-enhanced biodiesel can boost engine performance, with machine learning effectively predicting metrics. Despite higher costs, low concentrations and efficiency gains might justify their use in specialized applications. Commercial adoption needs a thorough cost–benefit analysis.

## Supplementary Material



## Notes

The 2D contour plots of XGBoost BTE and BSFC for training and testing instances (PDF)
