# Supplementary material for: Enhancing Engine Performance and Sustainability: Gold Nanoparticles and Machine Learning for Biodiesel Optimization in Compression Ignition Systems
Source: ACS Omega. 2025 Oct 3;10(40):46634–47. doi: 10.1021/acsomega.5c03571 (PMC12529385; doi:10.1021/acsomega.5c03571)
Supplement: Supplementary file 1 [file ao5c03571_si_001.pdf]

Enhancing Engine Performance and Sustainability: Gold Nanoparticles and Machine Learning for Biodiesel Optimization in Compression Ignition Systems

Amith Gadagi[1], Sneha Bandekar[2], Santhosh Paramasivam[3,*], Umesh Basanagouda Deshannavar[4,*], Natarajan Rajamohan[5,*], Chandrashekar Adake[1], Prasad G. Hegde[2], Gianluca Gatto[3]

[1]Department of Mechanical Engineering, KLE Technological University's Dr. M. S. Sheshgiri College of Engineering and Technology, Belagavi, India - 590008

[2]Department of Chemical Engineering, KLE Technological University's Dr. M. S. Sheshgiri College of Engineering and Technology, Belagavi, India - 590008

[3]*Department of Electrical and Electronic Engineering, University of Cagliari, Italy – 09123.*

[4]Department of Chemical Engineering, Tatyasaheb Kore Institute of Engineering and Technology, Warananagar, India - 416113

[5]Chemical Engineering Section, Faculty of Engineering, Sohar, Sohar University, Oman.

[*]Corresponding authors: Santhosh Paramasivam (santhosh.paramsivam@unica.it); Natarajan Rajamohan (rnatarajan@su.edu.om); Umesh Basanagouda Deshannavar (deshannavar@gmail.com)

Supplementary Information

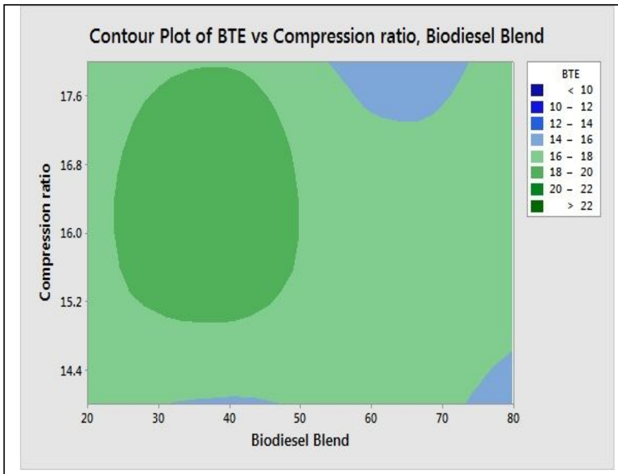

(a)

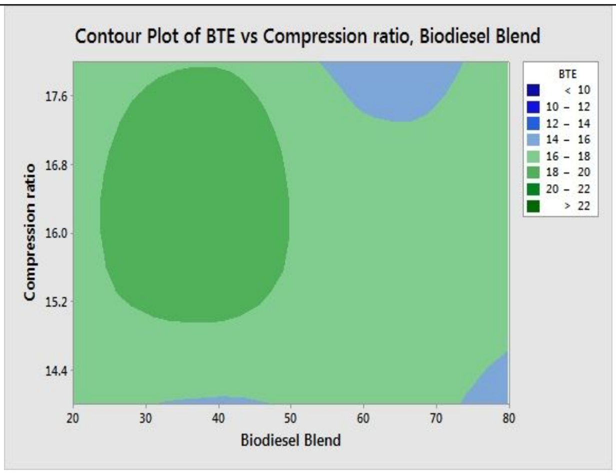

(b)

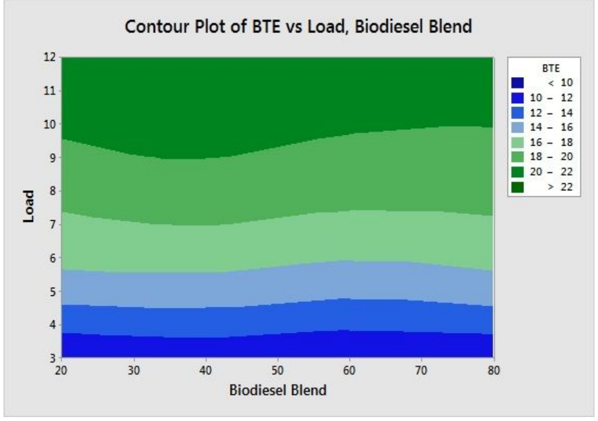

(a)

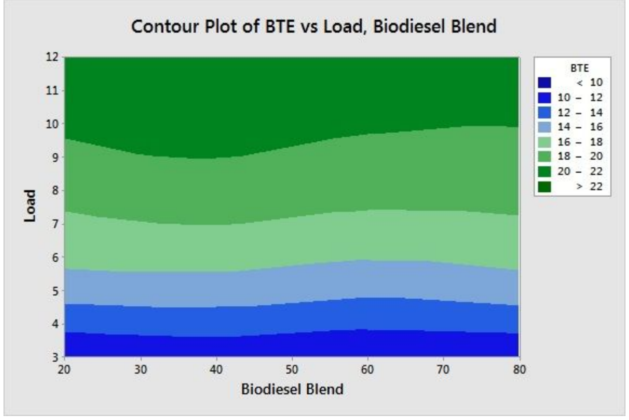

(b)

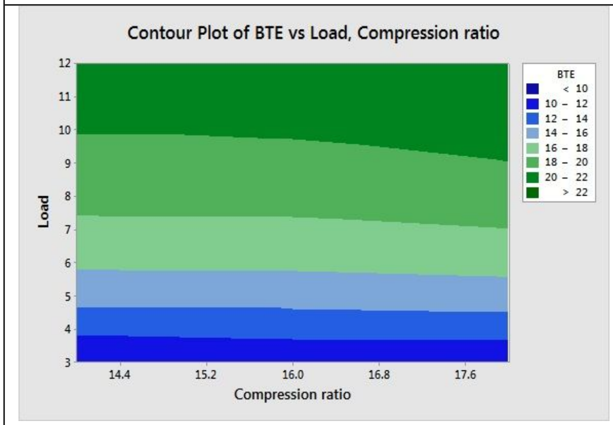

(a)

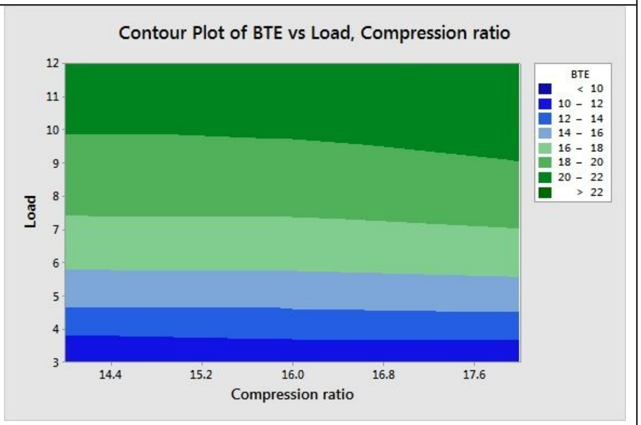

(b)

Figure S1 Comparison plot of BTE (%) Training dataset: a) XGBoost, b) Experimental [BTE (%), Load (kg), Biodiesel Blend (% of Biodiesel)]

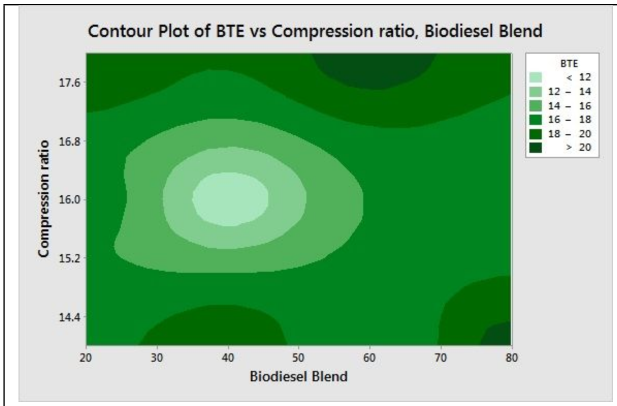

(a)

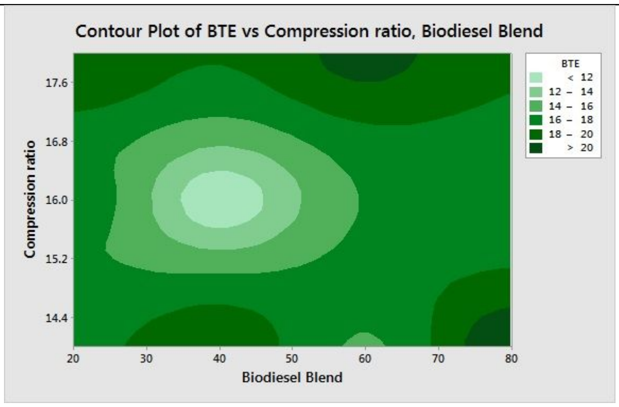

(b)

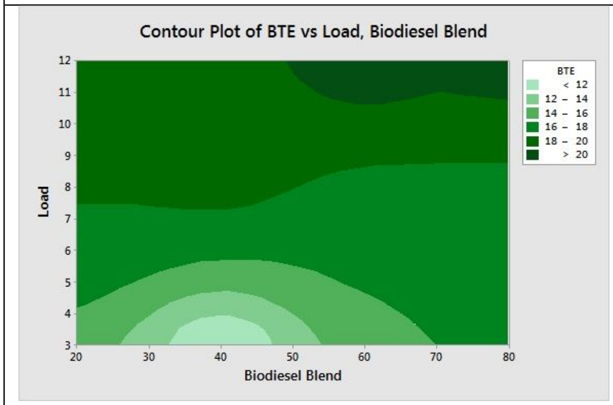

(a)

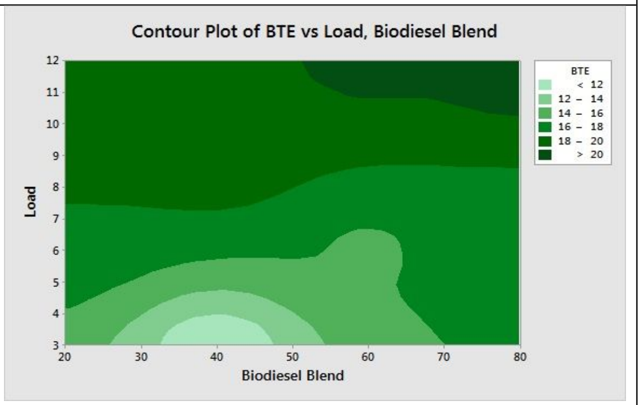

(b)

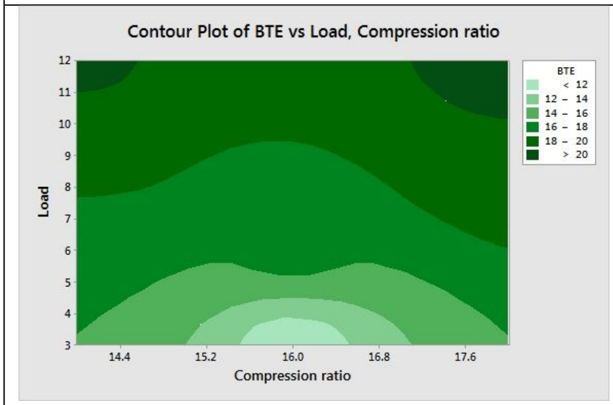

(a)

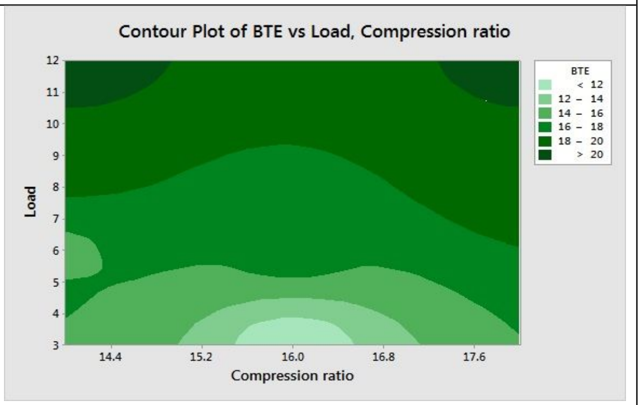

(b)

Figure S2 Comparison plot of BTE (%) Testing dataset: a) XGBoost, b) Experimental [BTE (%), Load (kg), Biodiesel Blend (% of Biodiesel)]

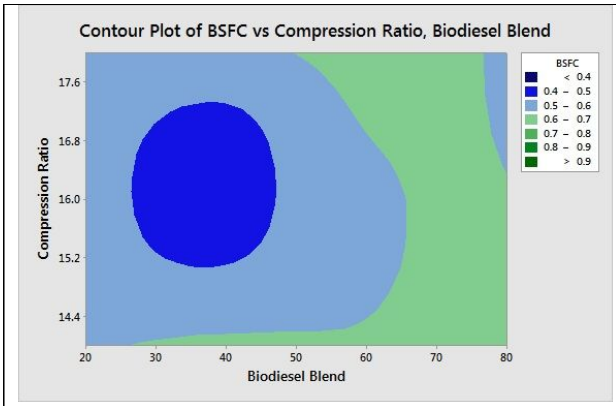

(a)

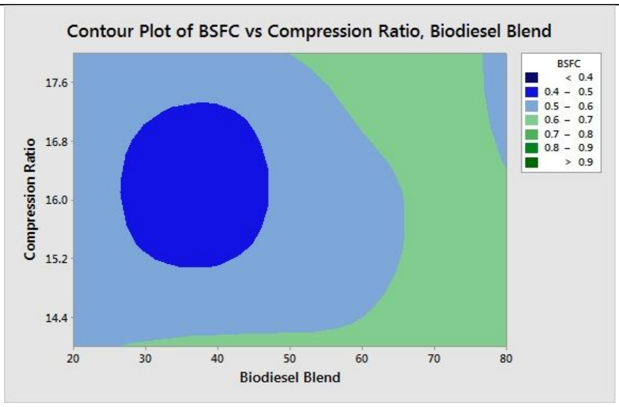

(b)

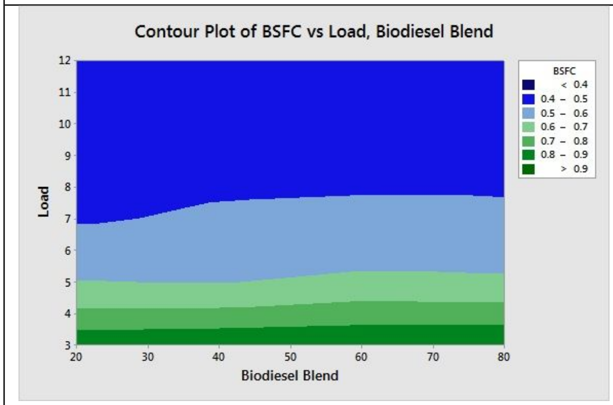

(a)

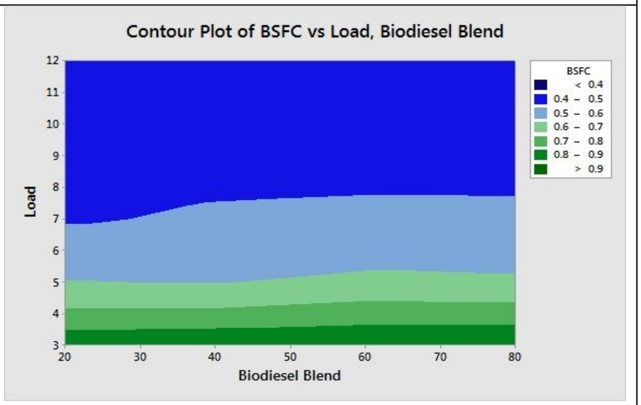

(b)

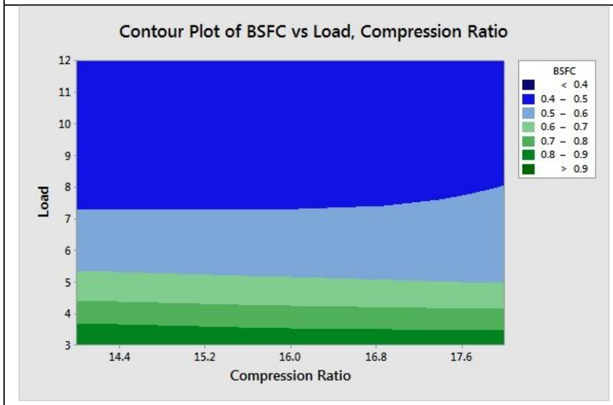

(a)

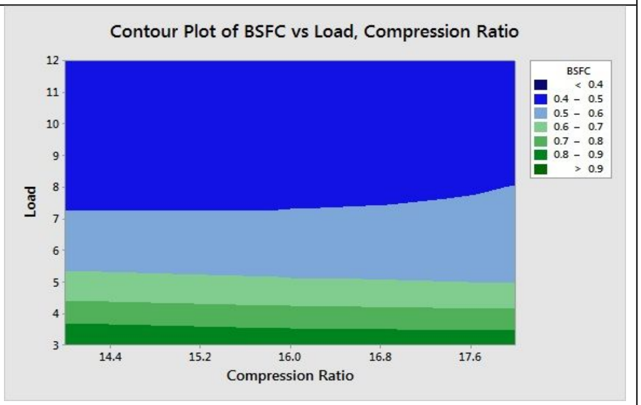

(b)

Figure S3 Comparison plot of BSFC (kg/kW-hr) Training dataset: a) XGBoost, b) Experimental [BSFC (kg/kW-hr), Load (kg), Biodiesel Blend (% of Biodiesel)]

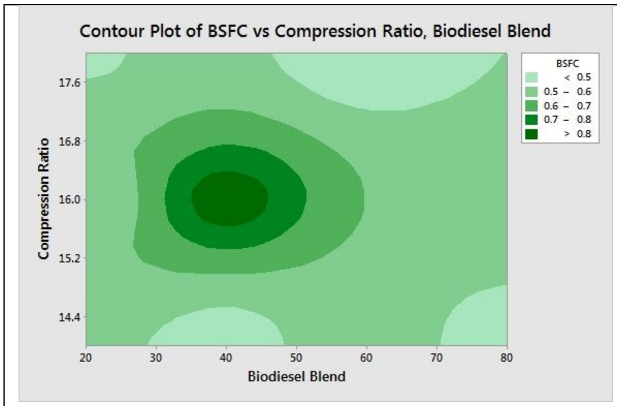

(a)

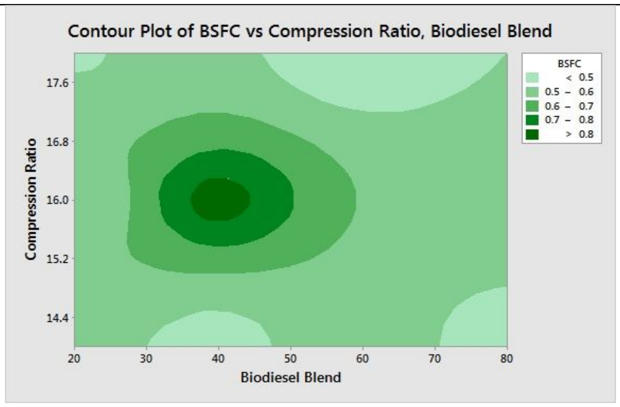

(b)

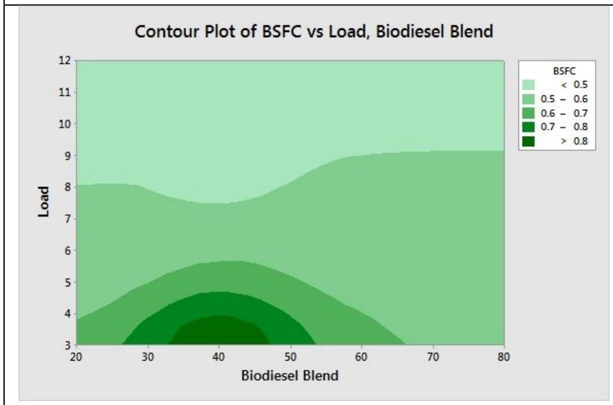

(a)

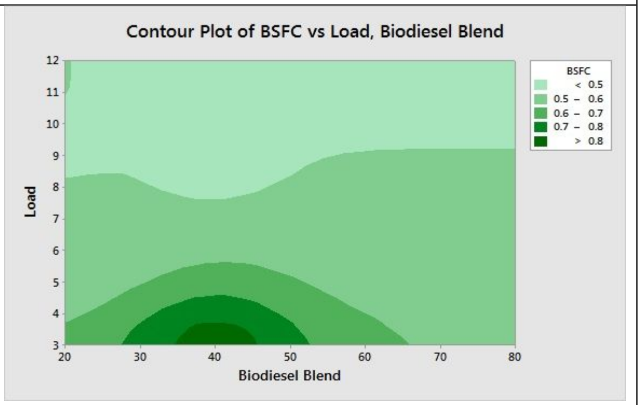

(b)

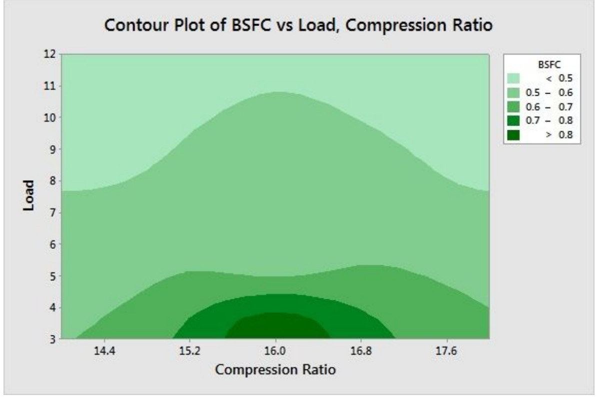

(a)

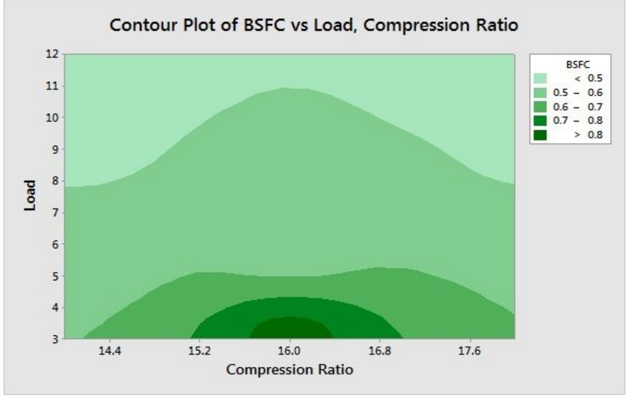

(b)

Figure S4 Comparison plot of BSFC (kg/kW-hr) Testing dataset: a) XGBoost, b) Experimental [BSFC (kg/kW-hr), Load (kg), Biodiesel Blend (% of Biodiesel)]
